# pH analysis of still and carbonated bottled water: Potential influence on dental erosion

**DOI:** 10.1002/cre2.535

**Published:** 2022-02-21

**Authors:** Mariana Morgado, Carla Ascenso, Joana Carmo, José João Mendes, Ana Cristina Manso

**Affiliations:** ^1^ Clinical Research Unit, Egas Moniz Higher Education School Centro de investigação interdisciplinar Egas Moniz (CiiEM) Caparica Portugal

**Keywords:** bottled water, dental erosion, erosive potential, pH

## Abstract

**Objective:**

To assess pH values to characterize bottled water in Portugal, being able to provide information for both patients and clinicians about its erosive potential, as a tool to prevent the ingrowing prevalence of dental erosion and its progression, especially in patients who are at greater risk, such as those with dry mouth syndrome, making the dissemination of this knowledge a fundamental tool for clinical decision.

**Materials and Methods:**

One hundred and five common brands of bottled water (*n* = 105), commercialized in Portugal, were analyzed. Of these, 73 were smooth water (Group A) and 32 carbonated water (Group B). All pH values were assessed by potentiometric measurement with a calibrated electrode. For each brand, five independent measurements were recorded at 25°C for further calculation of the mean pH value and standard deviation.

**Results:**

Focusing on the mean pH values from Group A, one had a pH mean value lower than 5.2, four between 5.2 and 5.5, thirty‐seven between 5.5 and 6.8, and thirty‐one higher than 6.8. In Group B, ten had a mean pH value lower than 5.2, ten between 5.2 and 5.5, twelve between 5.5 and 6.8, and none above 6.8.

**Conclusions:**

Bottled water, commercialized in Portugal, has different mean pH values, some below the critical threshold of enamel and/or dentin, suggesting that they may have a greater risk of consumption than others, only with respect to the pH parameter of erosive potential. Further investigation concerning this area is needed for wider conclusions.

## 1 INTRODUCTION

Dental erosion, or dental wear of erosive etiology, has become increasingly significant in the long‐term health of the dentition and well‐being of those who suffer from its effects, despite the lack of awareness of its importance (Ravichandran et al., 2018), with the differential diagnosis remaining to be a challenge for professionals in the field of dental medicine (Ganss & Schlueter, 2017).

The knowledge of the erosive potential of beverages is essential for the development of preventive strategies in patients with clinical erosion (Wright, 2015). The critical pH below which demineralization is expected to occur is represented by a value below the range between 5.2 and 5.5 in enamel, whereas in root dentine it is expected to be under 6.8 (Delgado et al., 2018), Nonetheless, many of the drinks described in the literature are compatible with pH values less than or equal to 4 and the solubility of apatite is considered to be compromised to this value under conditions to which buffers must be added, both in models and in ex vivo studies (Dorozhkin, 2012; Larsen et al., 1993). The study of monitoring the buffering effect in each drink is also documented, although this was not our aim, as the pH of mineral water drinks varies around 5 and above and their buffering is generally poor. As the solubility of enamel apatite is greatly increased, only beverages with a pH above 3.5 can realistically benefit from calcium phosphate supplementation in neutralizing enamel dissolution (Larsen et al., 1993); the factors known to cause erosion include all types of acidic foods with a low concentration of calcium and phosphate (Nayak et al., 2019; Zero, 1996).

The relationship of a variety of chemical properties of several products with their erosive potential has mainly focused on their pH level, buffer capacity, degree of saturation, calcium concentration, phosphate, and erosion inhibitors, such as the fluoride ion, with evidence that pH level is the dominant factor in erosion (Barbour et al., 2011). Concerning data on studies with the beverage and simple acid solutions for the detection of erosion (weakening or loss of tooth tissue), most methods focus on the pH region surrounding the value 5.0. (Barbour et al., 2011). Erosion by liquids with a pH below 7.0 (maximum) can be demonstrated, despite requiring a high area/volume quotient (Reddy et al., 2017), periods of exposure from 4 to 7 days (Adhani, 2015), or measurement by nanoindentation (Barbour et al., 2003).

In healthy individuals, the pH of saliva varies between 6.20 and 7.60 (Al et al., 2018) and is maintained at a non‐harmful level to the oral cavity through three buffer systems (bicarbonate, phosphate, and protein) (Dawes et al., 2015), depending and being strongly associated with the salivary secretion flow (Aframian et al., 2006).

The reduction of salivary flow affects 15%–30% of the population in patients aged 20–69 years (Flink et al., 2008) being characterized by total or partial loss of saliva production caused by hypofunction of the salivary glands (Delgado & Olafsson, 2017), with higher prevalence in the geriatric population (Affoo et al., 2015). Several treatment strategies for xerostomia have been proposed with the aim of reducing patients' symptoms and/or increasing salivary flow (Zero, 1996).

The nonpharmacological pathway to alleviate xerostomia symptoms is based on water reinforcement, with a high frequency of water intake (Delgado & Olafsson, 2017). This reinforcement is mandatory in the relief of these patients symptoms since dryness of the oral cavity and thirst are covariables (Fukushima et al., 2019), leading to fluid intake behaviors that result in a decrease in dry mouth sensation (Becker et al., 2017) and provoke a sensation of relief. Consequently, given the increased production of saliva potentiated by the gradual ingestion of liquids, and subsequent satisfaction, the patient will be motivated to continue this drinking behavior (Machete, 2015). Recent studies have proven that rehydration plays a key role in the increase of salivary flow and maintenance of the salivary pH in neutral values (Villa et al., 2015).

In general, some individuals have the belief that some water, especially bottled water, is safer than tap water, when in reality it may be acidic and potentially harmful to the dentition (Lussi et al., 1993). Since there is no database concerning the chemical properties of bottled water commercialized in Portugal, and the presentation of the pH value and its respective measurement temperature on the packages' labels is not mandatory, our purpose is to assess such values to characterize bottled water in Portugal, and be able to provide information for both patients and clinicians about its erosive potential, especially to patients who are at greater risk, such as those with dry mouth syndrome, making the dissemination of this knowledge a fundamental tool for clinical decision and to the prevention of ingrowing prevalence of dental erosion and its progression.

## 2 MATERIALS AND METHODS

### 2.1 Sample collection

One hundred and five common brands of bottled water, five of each brand, were analyzed to perform five recordings *per* water brand. These were purchased in the greater Lisbon and Tejo valley area, in large surfaces, as well as, other small local trading surfaces. Each type and water brand were purchased at the same location.

Of the 105 water brands, 73 were still water (365 water bottles) and 32 carbonated water (160 water bottles). As a result, 525 bottled water samples were tested. Of the 73 still water brands, 28 were mineral water (140 water bottles) and 45 were spring water (225 water bottles). From the 32 carbonated water brands (160 water bottles), 16 were gasified (80 water bottles) and the same number gasocarbonated water brands (80 water bottles). Among the 16 gasified water brands (80 water bottles), 13 originated from mineral natural (65 water bottles) and 3 of spring origin (15 water bottles).

During this study, all bottled water were stored at room temperature.

The water bottles were divided into two groups, depending on their type: Still Water (Group A) and Carbonated Water (Group B). Group A (Tables 1 and 2) was formed by 73 common brands of bottled still water, of national commercialization, while Group B (Table 3) had 32 common brands of carbonated bottled water, of national commercialization.

**Table 1:** Detailed pH measurements of Group A's samples—Bottled Still Water brands, commercialized in Portugal, with pH < 5.2, 5.2 < pH < 5.5 and 5.5 < pH < 6.8

Water brand	pH Sample 1	pH Sample 2	pH Sample 3	pH Sample 4	pH Sample 5	pH mean value	Standard deviation
7 FONTES®	5.15	5.08	4.98	4.93	4.95	5.02	0.09
ÁGUA SÃO MARTINHO®	5.30	5.37	5.31	5.21	5.23	5.28	0.06
ROCK WATER®	5.32	5.32	5.33	5.36	5.32	5.33	0.02
DIA®	5.54	5.44	5.41	5.42	5.36	5.43	0.07
SALUTIS®	5.52	5.48	5.51	5.45	5.53	5.50	0.03
VITALIS®	5.52	5.51	5.54	5.55	5.46	5.52	0.04
ARO®	5.69	5.72	5.70	5.65	5.66	5.68	0.03
LUSO®	5.67	5.69	5.70	5.66	5.72	5.69	0.02
SERRABRAVA®	5.78	5.80	5.78	5.75	5.77	5.78	0.02
CALDAS DE PENACOVA®	5.85	5.78	5.80	5.80	5.79	5.80	0.03
UP®	5.93	5.92	5.87	5.90	5.88	5.90	0.03
MARCA GUIA®	6.05	5.95	6.00	5.81	5.78	5.92	0.12
SERRANA®	6.02	5.91	5.91	5.91	5.87	5.92	0.06
FONTE DE AMORES®	5.83	5.85	5.95	6.03	6.02	5.94	0.09
CELEIRO®	6.20	5.94	5.94	5.81	6.12	6.00	0.16
FASTIO®	5.96	5.95	6.13	6.08	5.97	6.02	0.08
NESTLÉ® AQUAREL	6.01	6.11	6.01	6.00	5.96	6.02	0.06
PINGO DOCE® ‐ SERRA	6.03	6.06	6.11	6.10	6.16	6.09	0.04
ALCAFAZ®	6.17	6.13	6.11	6.11	6.15	6.13	0.03
NATURIS®	6.29	6.18	6.08	6.14	6.18	6.17	0.08
ALARDO®	6.26	6.15	6.08	6.14	6.27	6.18	0.08
MILFONTES®	6.17	6.17	6.16	6.17	6.24	6.18	0.03
CONTINENTE®	6.33	6.34	6.35	6.32	6.36	6.34	0.02
A PADARIA PORTUGUESA®	6.44	6.39	6.33	6.35	6.32	6.37	0.05
PINGO DOCE® ‐ FH6	6.67	6.45	6.42	6.37	6.40	6.46	0.12
SPRING PORTUGAL®	6.49	6.51	6.48	6.45	6.49	6.48	0.02
SERRA DA ESTRELA®	6.58	6.28	6.46	6.54	6.64	6.50	0.14
CARAMULO®	6.44	6.50	6.52	6.54	6.56	6.51	0.05
SERRAS DE FAFE®	6.52	6.57	6.50	6.54	6.53	6.53	0.03
PINGO DOCE® ‐ Nascente GLACIAR	6.50	6.56	6.55	6.54	6.57	6.54	0.03
NUMEN®	6.53	6.54	6.54	6.56	6.56	6.55	0.01
VIMEIRO® LISA	6.57	6.57	6.54	6.57	6.55	6.56	0.01
SERRA DA PENHA®	6.57	6.49	6.53	6.68	6.56	6.57	0.07
AUCHAN®	6.76	6.69	6.65	6.55	6.64	6.66	0.08
FONTE DA NATUREZA®	6.64	6.66	6.65	6.68	6.69	6.66	0.02
SPA WATER®	6.73	6.69	6.63	6.66	6.62	6.67	0.05
LOS RISCOS®	6.44	6.86	6.69	6.82	6.70	6.70	0.16
FEATHER WATER®	6.74	6.73	6.71	6.72	6.74	6.73	0.01
CARVALHELHOS®	6.64	6.70	6.85	6.72	6.76	6.73	0.08
RIK&ROK®	6.58	6.72	6.76	6.79	6.93	6.76	0.13
VOLVIC®	6.51	6.79	6.90	6.86	6.78	6.77	0.15
HEALSI®	6.81	6.80	6.78	6.78	6.77	6.79	0.02

**Table 2:** Detailed pH measurements of Group A's samples—Bottled Still Water brands, commercialized in Portugal, with pH > 6.8

Water brand	pH Sample 1	pH Sample 2	pH Sample 3	pH Sample 4	pH Sample 5	pH mean value	Standard deviation
FONTES DE LÁ®	6.82	6.93	6.79	6.79	6.77	6.82	0.06
EARTH WATER®	7.05	6.98	7.04	6.88	6.88	6.97	0.08
TOP BUDGET®	7.13	7.09	7.07	7.10	7.08	7.09	0.02
PERDIZ®	7.11	7.14	7.14	7.17	7.16	7.14	0.02
CONTREX®	7.05	7.20	7.22	7.22	7.13	7.16	0.07
MARQUE REPERE®	7.19	7.18	7.21	7.20	7.19	7.19	0.01
HÉPAR®	7.19	7.23	7.21	7.20	7.24	7.21	0.02
VALE VELHO®	7.26	7.23	7.22	7.22	7.20	7.23	0.02
NUTRIENDI®	7.38	7.25	7.26	7.16	7.20	7.25	0.08
EVIAN®	7.19	7.26	7.33	7.34	7.32	7.29	0.06
VIMEIRO® ORIGINAL	7.32	7.33	7.35	7.36	7.37	7.35	0.02
SÃO SILVESTRE®	7.34	7.33	7.38	7.42	7.42	7.38	0.04
BIO SYNERGY® STILL	7.31	7.42	7.52	7.33	7.47	7.41	0.09
AMANHECER®	7.63	7.51	7.56	7.34	7.43	7.49	0.11
TIGER®	7.45	7.52	7.53	7.48	7.53	7.50	0.04
VITTEL®	7.49	7.60	7.60	7.58	7.56	7.57	0.05
ECO +®	7.59	7.57	7.60	7.62	7.61	7.60	0.02
SOLAN DE CABRAS®	7.68	7.65	7.70	7.67	7.68	7.68	0.02
MONTE PINOS®	7.65	7.67	7.70	7.72	7.71	7.69	0.03
APTONIA® SOURCE AMANDA	7.74	7.72	7.75	7.75	7.76	7.74	0.02
LUCHON®	7.78	7.74	7.75	7.77	7.76	7.76	0.02
FIJI®	7.75	7.79	7.80	7.79	7.78	7.78	0.02
FONTE DA FRAGA®	7.92	7.87	7.88	7.85	7.89	7.88	0.03
JANA®	7.91	7.89	7.92	7.89	7.90	7.90	0.01
APTONIA® ROCHE DES ECRINS	8.10	7.83	7.98	7.97	7.99	7.97	0.10
CRUZEIRO®	8.09	8.12	8.10	8.14	8.14	8.12	0.02
FONT VELLA®	8.10	8.15	8.16	8.20	8.21	8.16	0.04
MONT BLANC®	8.40	8.49	8.49	8.49	8.48	8.47	0.04
ACQUA PANNA®	8.64	8.64	8.67	8.69	8.69	8.67	0.03
CHIC®	8.78	8.82	8.86	8.88	8.88	8.84	0.04
MONCHIQUE®	9.62	9.64	9.59	9.61	9.61	9.61	0.02

**Table 3:** Detailed pH measurements of Group B's samples—Bottled Carbonated Water brands, commercialized in Portugal with pH < 5.2, 5.2 < pH < 5.5 and 5.5 < pH < 6.8

Water brand	pH Sample 1	pH Sample 2	pH Sample 3	pH Sample 4	pH Sample 5	pH mean value	Standard deviation
SASKIA®	4.23	4.22	4.22	4.20	4.22	4.22	0.01
LUSO®	4.68	4.72	4.71	4.73	4.70	4.71	0.02
CARVALHELHOS®	4.82	4.83	4.81	4.80	4.82	4.82	0.01
ÁGUA CASTELLO®	4.95	5.04	5.05	4.97	5.10	5.02	0.06
VIMEIRO® SPARKLE	5.01	5.01	4.99	5.10	5.04	5.03	0.04
SAN PELLEGRINO®	5.05	5.08	5.02	5.09	5.11	5.07	0.04
PERRIER®	4.92	5.07	5.13	5.17	5.16	5.09	0.10
SAGUARO®	5.09	5.12	5.11	5.22	5.17	5.14	0.05
MARQUE REPERE®	5.17	5.15	5.15	5.15	5.13	5.15	0.01
ÁGUA CASTELLO® FINNA	5.16	5.18	5.13	5.19	5.10	5.15	0.04
AUCHAN®	5.18	5.20	5.23	5.26	5.20	5.21	0.03
DIA®	5.28	5.21	5.27	5.28	5.22	5.25	0.03
CONTINENTE®	5.29	5.26	5.26	5.27	5.28	5.27	0.01
MIL FONTES® SLIGHTLY GASIFIED	5.31	5.27	5.30	5.31	5.32	5.30	0.02
VIMEIRO®	5.31	5.31	5.34	5.37	5.37	5.34	0.03
ECO +®	5.31	5.35	5.35	5.34	5.36	5.34	0.02
ARIEIRO®	5.34	5.34	5.34	5.38	5.37	5.35	0.02
MIL FONTES® GASIFIED	5.33	5.34	5.35	5.37	5.40	5.36	0.03
LOOK®	5.35	5.40	5.43	5.42	5.44	5.41	0.04
BIO SYNERGY® SPARKLING	5.42	5.42	5.42	5.39	5.45	5.42	0.02
CAMPILHO®	5.70	5.72	5.76	5.74	5.73	5.73	0.02
VICHY CATALAN®	5.73	5.70	5.78	5.70	5.80	5.74	0.05
ST. DIERY®	5.75	5.77	5.77	5.81	5.83	5.79	0.03
BADOIT®	5.81	5.80	5.81	5.83	5.85	5.82	0.02
PEDRAS SALGADAS®	5.99	5.95	5.90	5.90	5.97	5.94	0.04
FRIZE®	5.90	6.04	5.96	5.96	5.99	5.97	0.05
PINGO DOCE® ‐ BEMSAÚDE	5.99	6.00	6.04	5.97	6.04	6.01	0.03
VIDAGO®	6.03	6.07	6.02	6.00	6.03	6.03	0.03
AMANHECER®	6.02	6.02	6.00	6.09	6.10	6.05	0.05
PEDRAS SALGADAS® SLIGHTLY GASIFIED	6.19	6.17	6.22	6.22	6.22	6.20	0.02
VICHY CELÉSTINS®	6.28	6.24	6.30	6.33	6.33	6.30	0.04
ST. YORRE®	6.49	6.50	6.50	6.54	6.54	6.51	0.02

### 2.2 pH calibration and analysis

Both groups were analyzed for their pH value by direct potentiometric measurement with CRISON® Basic 20 pH meter with an ORP Sension + pH3 electrode with resolution 0.01 pH, 1 mV, 0.1°C and measurement error (±1 digit) ≤0.01 pH, ≤1 mV, and ≤0.2°C. This electrode was calibrated in the pH range of 4.00–9.00, by a three‐pH calibration method, with standard buffer solutions, at room temperature. For each water brand, five independent pH measurements were recorded at 25°C. Results were organized into four categories: pH < 5.2, pH between 5.2 and 5.5, pH between 5.5 and 6.8, and pH>6.8.

### 2.3 Statistical analysis

Starting from the values obtained from the pH value measurements of the pentaplicates by brand, the mean values and respective standard deviation (SD) was calculated, using Microsoft® Excel for Mac 2019, version 16.29.1.

## 3 RESULTS

All pH data were expressed as mean value ± SD.

**Figure 1:**
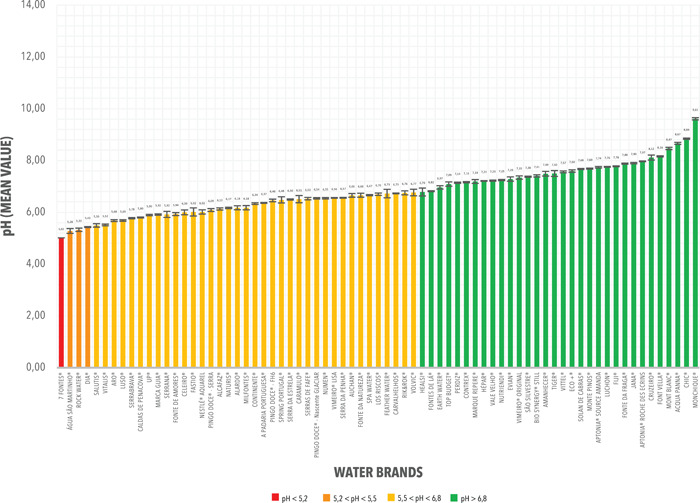
pH mean values and corresponding standard deviations (SD) from the measurements registered from Group A. Bottled Still Water Brands commercialized in Portugal

Seventy‐three bottled still water brands (365 samples), corresponding to Group A, had pH range values of 5.02 ± 0.09 and 9.61 ± 0.02 (Figure 1), and a mean (SD) value of 6.81 ± 0.05.

In Group A, most bottled still water brands had a pH mean value between 5.5 and 6.8 (37 of 73; 50.68%). 42.47% presented mean pH values greater than 6.8 (31 of 73), 5.48% values between 5.2 and 5.5 (4 of 73), and 1.37% values below 5.2 (1 of 73). The detailed pH measurements for Group A are as shown in Tables 1 and 2.

**Figure 2:**
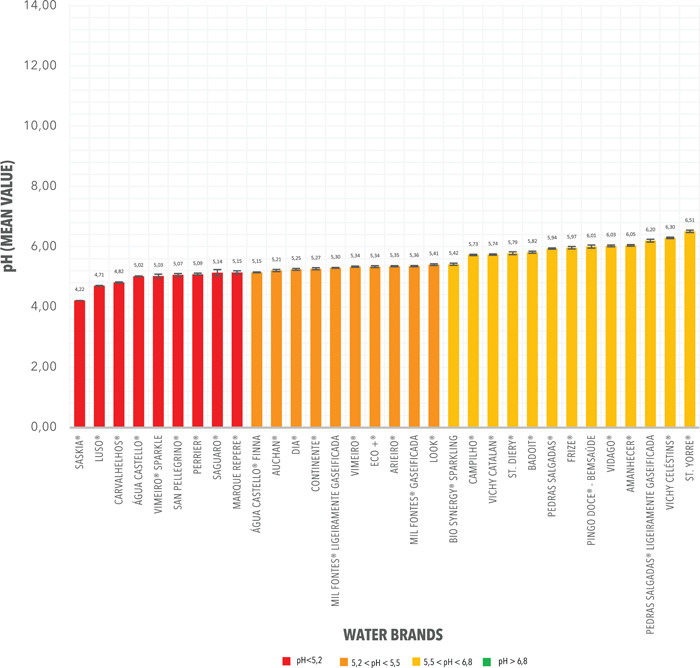
pH mean values and corresponding standard deviations (SD) from the measurements registered from Group B. Carbonated Water Brands commercialized in Portugal

Thirty‐two bottled carbonated water brands (365 samples), corresponding to Group B, had pH range values of 4.22 ± 0.01 and 6.51 ± 0.02 (Figure 2), and a mean (SD) value of 5.46 ± 0.03.

In Group B, most bottled carbonated water brands had a pH mean value between 5.5 and 6.8 (12 of 32; 37.50%). 31.25% presented mean pH values between 5.2 and 5.5 (10 of 32) and 31.25% values below 5.2 (10 of 73). There were no values greater than 6.8. The detailed pH measurements for Group B are shown in Table 3.

Most bottled water brands tested had a pH mean value within the range of 5.5 and 6.8 (49 of 105; 46.67%). 29.52% presented mean pH values greater than 6.8, 13.33% values between 5.2 and 5.5% and 9.52% values below 5.2.

## 4 DISCUSSION AND CONCLUSIONS

According to studies carried out in other countries, still water has a minimal erosive potential in dental enamel and synthetic hydroxyapatite, while carbonated water presents slightly higher dissolution (Reddy et al., 2017). Recent studies have concluded that chronic exposure to acidic water can cause tooth demineralization and result in dental erosion (Adhani, 2015).

A previous study conducted in Portugal (Parry et al., 2001) registered that the bottled still water brands of national consumption had described pH values ranged between 5.5 and 9.4, while for carbonated water brands the mean of the pH values varied between 5.3 and 6.5 (Parry et al., 2001). In this case, we considered the pH of water in a range between 5.02–9.62, for still water and 4.23–6.49 for carbonated water, not addressing the other variables, in addition to pH, with none of the water brands showing a pH of 4. The difference in values is mainly due to the fact that the tested water brands and sample size were different.

In our study, we are concerned with characterizing the pH of these waters, in relation to patients who, as nonpharmacological therapy, adopt water reinforcement through water intake and who often do so due to hyposialia and xerostomia events, without the clinician knowing what is the best water to prescribe. The fact is that, in these cases, patients' saliva is quantitatively altered, at least, and its buffering capacity very likely too.

We are aware that the literature describes that apatite can be compromised at a pH of 4, taking into account drinks compatible with this pH, where there is the presence of buffers. As a result of apatite dissolution, pH changes to higher values (Lussi et al., 1993) and the greater the buffering effect or acid concentration in the beverage, a higher amount of apatite will be dissolved before the pH approaches neutral and the dissolution ends (Leib et al., 2016). Other authors claim that brushite precipitation exerts a masking and competitive effect as the apatite dissolves, which will prevent solution saturation and thus maintain a continuous enamel apatite dissolution for almost as long as the pH is so low; that is, the solubility of apatite increases more than that of brushite below pH 4 (Larsen & Nyvad, 1999; Leib et al., 2016).

In the present study, 11 common brands commercialized in Portugal were identified as having a mean pH value below the critical threshold of the enamel of 5.2 with respect to the pH parameter of erosive potential. Of these, one belonged to Group A (still water brands) and ten to Group B (carbonated water).

At the dentin level, 75 common brands, commercialized in Portugal, were identified as mean pH value below the critical threshold of dentin, of 6.8. From these, 43 belonged to Group A (still water brands), whereas the other 32 belonged to Group B (carbonated water).

It has also been found that, when carbon dioxide is added to water from the same source, becoming carbonated, there is a decrease in the latter's mean pH value.

Considering that tooth erosion is multifactorial, depending on several variables for its progression, although drinks' pH is a dominant factor for the determination of its erosive potential, it is not unique, thus not representing the physiological conditions of dissolution, reflecting only the acidity or alkalinity characteristics of the samples. Despite the high number of samples and common brands studied, other areas of the country were not covered, where other bottled water brands existed, lacking a complete study of the bottled water of national commercialization. The systemic impact of the samples is also relevant since they may have a lower erosive dental potential but other harmful effects on the human body. In this case, and as the prescribed water is used for water reinforcement in patients suffering from hyposialia and/or xerostomia, we are faced with adverse conditions that are little referenced in literature and probably with insufficient biological buffers, such as adequate saliva from a quantitative and qualitative.

For the development of future studies, it will be relevant to cover a larger number of samples, also considering the waters of the national public network compared to bottled and within these test flavored water, as well as effect on the dentition mimetizing physiological conditions by conducting pH cycles with quantification of fluorine, phosphate and calcium ions.

It will also be interesting to verify if there are significant differences in enamel and dentin dissolutions when exposed to carbonated water and slightly carbonated water. There is no database of the physico‐chemical characteristics of bottled water in Portugal available for consultation by the consumer or health professional, and therefore it is essential to establish one for the development of preventive strategies and patients' counseling at the dentistry department, especially those with greater erosion risk, such as patients suffering from xerostomia.

## CONFLICT OF INTERESTS

The authors listed certify that they have no affiliations with or involvement in any organization or entity with any financial or nonfinancial interest in the subject matter or materials discussed in this manuscript.

## AUTHOR CONTRIBUTIONS

*Conceptualization, methodology, and formal analysis*: M. Morgado, C. Ascenso, and C. Manso. *Resources*: M. Morgado and J. J. Mendes. *Data curation*: M. Morgado. *Investigation and writing—Original draft preparation*: M. Morgado. *Writing—Review and edition*: C. Ascenso, J. Carmo, J. J. Mendes, and C. Manso. *Supervision*: J. J. Mendes and C. Manso. All authors have read and agreed to the published version of the manuscript.

## Data Availability

The data that support the findings of this study are available from the corresponding author, M. M., upon reasonable request.
